# Overuse of Diagnostic Tests in Clinical Practice: Are Gynecologists Aware of the Scientific Guidelines?

**DOI:** 10.1055/s-0040-1713409

**Published:** 2020-07

**Authors:** Einstein Francisco Camargos, Valter Paz do Nascimento-Júnior

**Affiliations:** 1 Faculty of Medicine, Universidade de Brasília, Brasília, DF, Brazil

Dear Editor,

In the past decades, the technological arsenal available in the clinical practice to doctors has provided a significant increase in clinical propaedeutics. Advanced and expensive imaging tests (magnetic resonance imaging tests, computed tomography scans, ultrasound, and others) and modern blood tests (sex hormones, vitamins, serology tests, etc.) have allowed doctors to better diagnose and treat diseases such as syphilis, thyroid dysfunction, and cancer precociously. Many of these tests are over-requested in clinical practice for several reasons that go well beyond easy access.

On the other hand, there are a large number of international and national publications regarding the inappropriate and excessive use of technologies and tests.^1^ ^2^ As a result, testing is the single highest-volume medical activity, with an estimated up to 5 billion tests performed in the United States each year.^3^ Data from the Brazilian National Health Agency showed that between 2014 and 2015, the total of complementary exams reported reached 1.4 billion, with an estimated cost of more than 10 billion dollars.^4^ These costs impact not only the private system but the Brazilian National Health System (SUS) as well. Hence, payments to the doctors and SUS pay tables are decreasing over recent years in Brazil.

All medical specialties that request tests in the clinical practice are important to improve the proper use of these exams and to lower health costs. The gynecologists are the primary care physician to women and frequently request blood and image exams, greatly improving clinical diagnoses. However, we have observed in clinical routine—there are no studies about it—that many tests are requested inappropriately. For example, ultrasound imaging for assessment of thyroid nodules (in patients without symptoms who are undergoing evaluation for other medical complaints) or tumor markers for cancer diagnosis (in patients without symptoms). The US Preventive Services Task Force (USPSTF) does not recommend screening for thyroid nodules in adults without symptoms, because screening can result in the identification of indolent thyroid cancers, and treatment of these overdiagnosed cancers maybe increase the risk of patient harm.^5^ Other examples are serum tumor markers, such as CA-125 (cancer antigen 125), which is associated with ovarian cancer; carcino embryonic antigen (CEA), which is associated with colon cancer; carbohydrate antigen (CA 19-9), which is released by pancreatic cancer cells, and cancer antigen 15-3 (CA 15-3), which is used to monitor response to breast cancer treatment and disease recurrence.

Recently, we conducted a study (not yet published), in which we evaluated 6,878 users of private health insurance in Brasilia, Brazil, between 2010 and 2017. The rate of inappropriate exams (tumor markers) was surprisingly high (85%), considering the guidelines from the USPSTF,^6^ ^7^ ^8^ The Royal Australian College of General Practitioners (RACGP),^9^ and the American Society of Clinical Oncology (ASCO).^10^ Our results also showed that 9% of these inappropriately requested tumor markers tests were required by gynecologists, which was significantly higher than that requested by oncologists (*p* = 0.004). However, this was not a prerogative of gynecologists. General practitioners and cardiologists were amongst the ones who most requested exams too. In fact, this research was not designed to adress effectiveness between different medical specialties.

According to some studies, gaps in the physicians' knowledge of guidelines and concerns about misdiagnosis could be responsible for inappropriate exams request, even in the case of primary care physicians. According to a recent American study conducted with medical colleges of primary health care, it was shown that 1/3 of primary care doctors routinely experience uncertainty and challenges in ordering and interpreting diagnostic laboratory tests.^11^ The authors have commented on the manuscript that “improvement in information technology and clinical decision support systems and quick access to laboratory consultations may reduce physicians' uncertainty and mitigate these challenges”.

Thus, we would recommend that cientifical medical societies, including the Brazilian Federation of Gynecology and Obstetrics Associations, increase the number of publications addressing the correct use of tumor markers (and other exams) in clinical practice. An educational approach combined with feedback on utilization can reduce the number of laboratory tests unjustifiably requested.

Finally, it is imperative to recommend and remember that an accurate physical exam is needed before ordering blood tests. We must be careful with predefined exam lists that are printed using a simple touch of a button in the computer. In addition, the consequences after a false-positive result can be disastrous. Psychosocial distress, anxiety, and worries about cancer have been frequently observed in the physician's office. Furthermore, a “cascade” diagnostic approach after a false-positive initial test can trigger other implications.
